# *In Vitro* and *in Vivo* Effects of Three Different *Mitragyna speciosa* Korth Leaf Extracts on Phase II Drug Metabolizing Enzymes—Glutathione Transferases (GSTs)

**DOI:** 10.3390/molecules15010432

**Published:** 2010-01-20

**Authors:** Juzaili Azizi, Sabariah Ismail, Mohd Nizam Mordi, Surash Ramanathan, Mohd Ikram Mohd Said, Sharif Mahsufi Mansor

**Affiliations:** 1 Centre for Drug Research, Universiti Sains Malaysia, 11800 USM Penang, Malaysia; 2 School of Chemical Sciences and Food Technology, Universiti Kebangsaan Malaysia, 43600 Bangi, Selangor, Malaysia

**Keywords:** herb-drug interactions, glutathione transferases, *Mitragyna speciosa*, *in vitro*, *in vivo*

## Abstract

In the present study, we investigate the effects of three different *Mitragyna speciosa* extracts, namely methanolic, aqueous and total alkaloid extracts, on glutathione transferase-specific activity in male Sprague Dawley rat liver cytosol *in vitro* and *in vivo*. In the *in vitro* study, the effect of *Mitragyna speciosa* extracts (0.01 to 750 µg/mL) against the specific activity of glutathione transferases was examined in rat liver cytosolic fraction from untreated rats. Our data show concentration dependent inhibition of cytosolic GSTs when *Mitragyna speciosa* extract was added into the reaction mixture. At the highest concentration used, the methanolic extract showed the highest GSTs specific activity inhibition (61%), followed by aqueous (50%) and total alkaloid extract (43%), respectively. In *in vivo* study, three different dosages; 50, 100 and 200 mg/kg for methanolic and aqueous extracts and 5, 10 and 20 mg/kg for total alkaloid extract were given orally for 14 days. An increase in GST specific activity was generally observed. However, only *Mitragyna speciosa* aqueous extract with a dosage of 100 mg/kg showed significant results: 129% compared to control.

## 1. Introduction

*Mitragyna speciosa* Korth (*M. speciosa*) is found in tropical and subtropical regions of Asia and is categorized in the family Rubiaceae. The leaves of *M. speciosa* are often used to treat diarrhea or intestinal infections by amoeba and protozoa [1]. There is a report that the leaves have been abused for its addictive properties [2]. An indole alkaloid, mitragynine, was found to be the major constituent of *M. speciosa* leaves and is believed to be the contributor of the pharmacological effects of the plant. Our recent work [3] showed *M. speciosa* leaf extracts have antioxidant and antimicrobial activities. These pharmacological activities are often attributed to the alkaloids found in the leaf [4]. However, claims of its safe usage in folk medicine are unsubstantiated by scientific studies. An in-depth study of the interactions between herbs and drug metabolizing enzymes is essential to further delineate its use in various traditional medicinal practices

Glutathione transferases, GSTs (EC 2.5.1.18) are multifunctional enzymes capable of catalyzing the conjugation reaction between glutathione (GSH) and electrophilic compounds. These dimeric enzymes are found in cell in a variety of life forms [5] and are mostly involved in the detoxification of toxic and carcinogenic compounds in the cells, thereby protecting them against toxic injuries [6]. Moreover, GSTs also act as antioxidant enzymes due to their selenium-independent GSH peroxidase activity [7]. The amount of GSTs in the human liver is about 4–10% of total soluble proteins [8]. Due to their large quantities in the cell and broad substrate specificity, GSTs are to be expected to interact with novel compounds presented to them. In addition, GSTs activities have previously been shown to be modulated by plant products [9]. The objective of the present study was to investigate the effects of *M. speciosa* methanolic, aqueous and total alkaloid extracts on GSTs specific activity *in vitro* and *in vivo* in male Sprague Dawley rats.

## 2. Results and Discussion

This is the first and only study about the effect of *M. speciosa* extracts on ubiquitously found drug metabolizing and antioxidative enzymes, the glutathione transferases (GSTs). In this study, three different extracts of *M. speciosa* namely methanolic, aqueous and total alkaloid, were evaluated for their effects on GSTs. Most *M. speciosa* abusers in Malaysia consume the aqueous extract by boiling it in hot water. There may be some degradation specifically in the preparation of aqueous extracts, but no thorough investigation has been performed on all these extracts. However, this was not deemed critical since we are looking at the traditional method of preparing and consuming *M. speciosa*.

**Figure 1:**
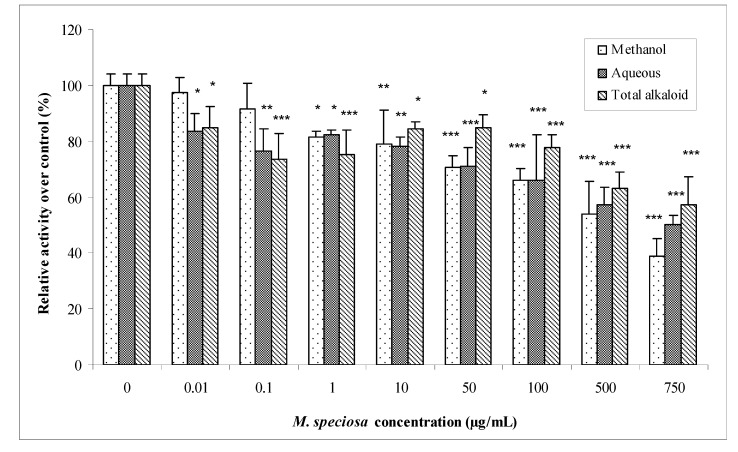
Percentage of GSTs specific activity compared with control for *in vitro* study. Values are mean ± SD for five determinations (n = 5). Statistical analysis was conducted using one way ANOVA and Dunnet test. * p < 0.05 *vs.* control (no *M. speciosa*), ** p < 0.01 *vs.* control (no *M. speciosa*), *** p < 0.001 *vs.* control (no *M. speciosa*).

The conventional reaction catalyzed by GSTs is the formation of a thioether between GSH and an electrophilic substrate. The reaction can be exemplified by using the probe substrate 1-chloro-2,4-dinitrobenzene (CDNB), which can react enzymatically or nonenzymatically with nucleophiles. Hence, the kinetics of thioether formation can be monitored over time and the activity can be calculated. Our *in vitro* study result show that addition of *M. speciosa* extracts into the reaction medium lead to concentration dependent inhibition of cytosolic GSTs in the male Sprague Dawley rat *in vitro*. The percentage inhibition of GSTs specific activity varied from 3–61% for methanolic extract, 16–50% for aqueous extract and 15–43% for total alkaloid extract. The IC_50_ values were calculated by plotting the percentage inhibition of GSTs specific activity versus log concentration of *M. speciosa* extracts. The analysis was done using GraphPad Prism^®^ 5 (Version 5.01, GraphPad Software, Inc., USA). The IC_50_ values for all extracts toward GSTs, however, were all greater than the highest concentration used (750 μg/ml). For these extracts, an accurate IC_50_ could not be determined because maximum inhibition (>70% inhibition) did not occur at higher doses [10]. Figure 1**.**

Plant phenolic compounds such as tannic acid, ellagic acid, ferrulic acid, caffeic acid, silybin, quercetin, curcumin and chlorogenic acid have been reported to be responsible for the inhibition of GSTs *in vitro* [11,12,13]. In our *in vitro* study, we used tannic acid as a positive control. Tannic acid showed 50% inhibition on GST specific activity at a concentration of 1.83 µg/mL or 1.08 µM (data not shown). This value coincided with a previous report of an IC_50_ of 1.78 µg/mL or 1.04 µM [13]. However not all plant phenols such as gallic acid and mellitic acid can inhibit GST specific activity [13]. It has been suggested that the presence of polyhydroxylations in plant polyphenols is important for GST inhibition [14]. In addition, flavonoids have been shown to inhibit GST activity in human blood platelets, rat liver and rat kidney [15]. Our recent publication [3] reported that *M. speciosa* leaf extracts have antioxidant and antimicrobial activities. Our published data [3] shown that *M. speciosa* extracts contain high amount of total phenolic content as follows: methanolic (105.6 mg of gallic acid equivalent/g extract) > total alkaloid (88.4 mg of gallic acid equivalent/g extract) > aqueous (66.0 mg of gallic acid equivalent/g extract). On the other hand, total flavonoid content is the highest in methanolic (91.1 mg of catechin equivalent/g extract) extract, followed by aqueous (28.2 mg of catechin equivalent/g extract) and total alkaloid extract (20.0 mg of catechin equivalent/g extract). Although total alkaloid extract shows a higher amount of total phenolic content than aqueous extract, it shows less total flavonoid content. This indicates that besides flavonoids, there are some other phenolic compounds present in the total alkaloid extract. Thus, our data shows that flavonoid is the main group of compounds that manifests GST inhibition *in vitro*. However, extrapolation of the data to humans need to be done carefully since rat liver isoenzymes of GSTs are known to be more sensitive to inhibition by plant phenols than human isoenzymes [16]. In the *in vivo* study, the treatment of the experiment animals with the three *M. speciosa* extracts for 14 days before sacrifice shows no significance difference in relative weight of liver to body weight (Table 1).

**Table 1:** Percentage liver weight to body weight index (%); no significant difference with the control.

Dose (mg/kg)	Percentage liver weight to body weight index (%)		
	Methanolic	Aqueous	Total alkaloid
0	3.40 ± 0.44	3.40 ± 0.44	3.40 ± 0.44
50	3.94 ± 0.64	3.50 ± 0.61	-
100	3.84 ± 0.26	3.00 ± 0.36	-
200	3.31 ± 0.67	3.19 ± 0.21	-
5	-	-	2.89 ± 0.43
10	-	-	3.12 ± 0.70
20	-	-	3.48 ± 0.40

In our preliminary toxicity study, we observed death of rats after treatment with 200 mg/kg total alkaloid extract. Therefore, we used 20 mg/kg dosage of total alkaloid extract to feed the rats. The similar dose for total alkaloid extract was used by Reanmongkol *et al.* in their study [17]. Our *in vivo* study using three dosages; low, medium and high dose, shows contradictory results, in which there is a general increase in specific activity compared to control (Table 2). Additionally, only rat liver cytosolic GSTs from rat treated with 100 mg/kg aqueous extract showed significant induction at p < 0.05 (129% GSTs specific activity compared with control).

Discrepancies between *in vitro* and *in vivo* results have been described elsewhere for antioxidant such as ellagic acid and curcumin that are *in vitro* inhibitors, but *in vivo* inducers of GSTs [16,18,19]. The induction might be due to the presence of compounds which increase the expression of GSTs *in vivo* which does not occur *in vitro* [20].

Plant extracts and GSTs interaction have been reported previously [9,21]. Induction of GSTs *in vivo* is potentially beneficial in protecting the cells from electrophilic insult. Electrophilic compounds are deficient in paired electrons and can result in several diseases, including cancer and neurodegenerative disorders, by snatching electrons from macromolecules such as DNA, proteins and lipids [22].

**Table 2:** Percentage of GSTs activity compared with control for *in vivo* study. Rats were treated with *M. speciosa* extract for 14 days before sacrifice. Values are mean ± SD for five determinations (n = 5). * p < 0.05 *vs*. control (no *M. speciosa*).

Dose (mg/kg)	Percentage of GSTs specific activity (%)		
	Methanolic	Aqueous	Total alkaloid
0	100 ± 15	100 ± 15	100 ± 15
50	101 ± 5	113 ± 16	-
100	121 ± 8	129 ± 13*	-
200	105 ± 7	111 ± 14	-
5	-	-	117 ± 7
10	-	-	95 ± 4
20	-	-	101 ± 9

Induction of GSTs could possibly result in increased protection against the toxic effects of eletrophilic chemicals or metabolites. In addition, GSTs also protect cells by sequestering compounds through their ability to bind most of the nonpolar and hydrophobic molecules noncatalytically (ligandin function). Their binding may inhibit the catalytic function of the GSTs [23]. However, some drugs are bioactivated by the GST catalytic pathway and rendered more toxic [24]. Moreover, overexpression of GSTs has been implicated in resistance to alkylating anticancer drugs [25], due to the ability of tumor cells to promote GSH conjugation catalyzed by GSTs [26]. On the other hand, potentially dangerous outcome may occur if GST-mediated scavenging of electrophilic xenobiotics is inhibited due to a reduced protection against electrophilic chemicals or metabolites [27].

## 3. Experimental

### 3.1. Chemicals

All reagents used were of analytical grade. 1-Chloro-2,4-dinitrobenzene (CDNB), glutathione-reduced formed (GSH), cupric sulfate pentahydrate, Folin & Ciocalteu’s phenol reagent, potassium sodium tartrate tetrahydrate and sodium carbonate were obtained from Sigma Chemicals Co., Ltd. (St. Louis, MO, USA). Propylene glycol (propane-1,2-diol) and polysorbate-80 (Tween-80) were purchased from Fisher Scientific (Loughborough, UK). Potassium dihydrogen orthophosphate was from Ajax Chemicals (New South Wales, Australia). Dipotassium hydrogen phosphate was from Riedel-de Haen (Seelze, Germany). Tannic acid was from R & M Chemicals (Canada). Potassium chloride was from BDH Chemicals Ltd (Poole, England).

### 3.2. Plant Collection

Fresh leaves of *M. speciosa* were obtained from Kedah, Malaysia. The plants were authenticated by a competent botanist at the Herbarium of the School of Biological Sciences, Universiti Sains Malaysia. The voucher specimen number of the plant is UKMB06509**.** The leaves were cleaned and oven dried at 40 °C for three days. Finally, the dried leaves were grinded to obtain in the powdered form.

### 3.3. Preparation of Plant Extracts

#### 3.3.1. Methanolic Extract

*M. speciosa* leaf powder (100 g) was Soxhlet extracted using AR grade methanol until the sample in the thimble become colourless. Finally the methanol was removed by rotary evaporator to give 20 g of crude methanolic extract and stored at −20 °C.

#### 3.3.2. Aqueous Extract

*M. speciosa* dried leaf powder (5 kg) was boiled in water (8 L) for 2 hours. Water was drained and the residue boiled for a second time under the same conditions. Finally the extract was blow dry to give 375 g of extract, which was stored at −20 °C.

#### 3.3.3. Total Alkaloid Extract

Dried and powdered leaves of *M. speciosa* (5 kg) were soaked in methanol for several days at room temperature. The suspension was filtered and the methanol was removed by rotary evaporator to give the crude methanolic extract. The extraction and evaporation procedure was repeated three times. Subsequently, 1 part of methanol extract was mixed with 35 parts of 90% acetic acid. The suspension was filtered and the filtrate washed with petroleum ether. The acidic layer is basified with sodium carbonate to pH 9 and extracted with chloroform for several times. The combined chloroform extract was dried over sodium sulphate and evaporated to give 5 g (0.5% yield) of crude alkaloid mixture.

### 3.4. Standardized extracts

All *M. specioasa* leaf extracts were standardized in reference to the content its bioactive compound mitragynine using validated HPLC assay method. The amount of mitragynine in methanolic, aqueous and total alkaloid extracts ranged from 6.0–8.0%, 0.1–0.5% and 22.0–24.0% respectively. The standardized methanolic, aqueous and total alkaloid leaf extracts were used for the *in vitro* and *in vivo* study.

### 3.5. Animals

Male Sprague Dawley rats (150–200 g) were obtained from the Animal House of Universiti Sains Malaysia (USM). The rats were maintained under controlled temperature (25 ± 2 °C), 12 h light/12 h dark conditions for one week before the start of the experiments. They were provided with water and food *ad libitium*. Animals were maintained and handled according to the recommendations of the USM Ethical Committee which approved the design of the animal experiments.

### 3.6. In Vitro Study

Ten rats were obtained and remain untreated. The rats were sacrificed by cervical dislocation and the livers were taken out for cytosolic fraction preparation as described in Section 3.7.

### 3.7. Preparation of Rat Liver Cytosolic Fraction

Rat livers were removed immediately after sacrifice was rinsed with ice-cooled distilled water followed by ice-cooled 67 mM potassium phosphate buffer (pH 7.4), blotted dry and weighed. Isolated rats liver samples were homogenized in 3 volumes of 67 mM potassium phosphate buffer (pH 7.4) containing 1.15% potassium chloride, using a Potter–Elvehjem homogenizer. After centrifugation of the homogenate fraction at 12,500× *g* for 20 minutes at 4 °C, the resultant supernatant was decanted to ultracentrifuge tubes (Optiseal^TM^) and centrifuge at 100,000× *g* for 60 min in a Optima^TM^ TLX refrigerated ultracentrifuge (Beckman Coulter, Inc., USA). The supernatant obtained was cytosolic fraction. Protein concentration was determined by the Lowry method [28]. The cytosolic fractions were stored at −80 °C until used.

### 3.8. In Vivo Study

Rats were randomly divided into 10 groups of six animals per each group. Three different dosages, 50 mg/kg, 100 mg/kg and 200 mg/kg were used. The dosage for total alkaloid extract is one tenth of the used dosage [17]. The methanol, aqueous and total alkaloid extract leaves were dissolved in cosolvent solution (propylene glycol-Tween 80-water = 4:1:4) and administered orally in a constant volume of 5 mL/kg [17]. Group 1 was control (received cosolvent); groups 2, 3 and 4 received methanolic extract (50, 100 and 200 mg/kg, respectively). Groups 5, 6 and 7 received aqueous extract (50, 100 and 200 mg/kg, respectively). Groups 8, 9 and 10 received total alkaloid extract (5, 10 and 20 mg/kg, respectively). All treatments were administered orally by oral gavages for 14 consecutive days. At the end of the treatments, the animals were sacrificed by cervical dislocation and the livers were collected for cytosolic fraction preparation.

### 3.9. GST Inhibition Assays

Inhibition of the activities of cytosolic GSTs by the plant extracts was assessed as described previously by Habig [29] with slight modifications. GST mediated conjugation of 1-chloro-2,4-dinitrobenzene (CDNB) to glutathione (GSH) was measured using a PlateCHAMELEON^TM^ multitechnology plate reader, 425-106 (Hidex Oy, Finland) at the wavelength of 340 nm for 5 minutes. Incubation mixtures (300 μL) contained 0.1 M potassium phosphate buffer pH 6.5, 30 mM CDNB, 30 mM GSH, and GST enzymes (0.125 mg/mL rat liver cytosolic fraction). The plant extracts were dissolved in distilled water were tested at a concentration range of 0.01–750 μg/mL. Tannic acid was used as a positive control for the *in vitro* study at a concentration range of 0.3–10 μg/mL. For the *in vivo* study, the cytosolic fractions from treated rats were used without the addition of plant extracts to the reaction mixture. All assays were linear functions of protein concentration and of time for at least 5 minutes. The enzyme activities were expressed as percent specific activity over control [26].

### 3.10. Statistical Analysis

All values are expressed as means ± standard deviation (SD) of five samples. ANOVA followed by Dunnet test was used to evaluate the significance differences of the results obtained. All computations were performed using GraphPad Prism^®^ 5 for windows software (Version 5.01, GraphPad Software, Inc., USA).

## 4. Conclusions

In conclusion, according to our data, *M. speciosa* extracts show in both *in vivo* and *in vitro* studies significant herb-drug interactions. However, the inhibition of GSTs *in vitro* is very slight as the IC_50_ values for all three extracts are more than the highest concentration (750 μg/mL) used. In *in vivo* study, only *M. speciosa* aqueous extract at a dose of 100 mg/kg show significant induction although all other extracts at different doses show general increase in GSTs specific activity. The inductive effect in rat *in vivo* study does not necessarily represent that which may occur in human, *in vivo*. Therefore, further research is necessary to investigate the clinical relevant of *M. speciosa* extracts-GSTs interactions in human body.
